# The HepG2 Cell Line as a Model for Studying Metabolic Dysfunction-Associated Steatotic Liver Disease

**DOI:** 10.3390/ijms27083399

**Published:** 2026-04-10

**Authors:** Anna Kotlyarova, Aleksandra Iskrina, Stanislav Kotlyarov

**Affiliations:** 1 Department of Pharmacy Management and Economics, Ryazan State Medical University, 390026 Ryazan, Russia; kaa.rz@yandex.ru; 2 Department of Nursing, Ryazan State Medical University, 390026 Ryazan, Russia

**Keywords:** MASLD, NAFLD, MASH, HepG2, free fatty acids, steatosis, lipotoxicity, 2D monocultures, 3D systems, spheroids, organ-on-a-chip, co-culture

## Abstract

Metabolic dysfunction-associated steatotic liver disease (MASLD), formerly known as nonalcoholic fatty liver disease (NAFLD), is the most common chronic liver disease in the world. The disease progresses from steatosis to metabolic dysfunction-associated steatohepatitis (MASH), fibrosis, cirrhosis, and hepatocellular carcinoma. The modern concept of “multiple parallel hits” interprets disease progression as the result of the synergistic action of lipotoxicity, oxidative stress, mitochondrial dysfunction, endoplasmic reticulum stress, proinflammatory signals, and gut–liver axis dysfunction. Against the background of the limited translation of preclinical data from animal models due to interspecies differences, the importance of human-oriented in vitro platforms compatible with controlled design and high-throughput screening is increasing. The current review analyzes MASLD models based on the HepG2 cell line, systematizing steatosis induction protocols, evaluating the metabolic characteristics and limitations of this cell, and comparing 2D monocultures, 3D systems, and co-cultures. HepG2 has been shown to demonstrate a predictable steatogenic response to free fatty acids (FFAs) and is convenient for reproducing early stages of pathogenesis and primary pharmacological selection of compounds. At the same time, key limitations of the model are highlighted, namely tumor origin, glycolytic shift (Warburg effect), reduced β-oxidation, impaired very-low-density lipoprotein (VLDL) assembly and secretion, and sharply reduced cytochrome P450 (CYP450) activity, as well as limited reproducibility of fructose-induced de novo lipogenesis (DNL). Comparative analysis demonstrates an increase in physiological relevance with the transition from 2D to 3D and multicomponent co-cultures, accompanied by increased complexity and cost, but allowing for the modeling of inflammation and fibrogenesis. The review justifies approaches to selecting the appropriate platform based on the specific research task.

## 1. Introduction

Nonalcoholic fatty liver disease (NAFLD), or metabolic dysfunction-associated steatotic liver disease (MASLD) according to the new classification [1,2], is the leading cause of chronic liver disease worldwide, with a prevalence of more than 30% among the adult population [3,4,5,6,7,8], which continues to increase [9,10,11]. The term MASLD more accurately reflects the metabolic systemic context of the disease than NAFLD. The MASLD spectrum includes simple steatotic infiltration, metabolically associated steatohepatitis (MASH, formerly nonalcoholic steatohepatitis (NASH)), progressive fibrosis, cirrhosis, and hepatocellular carcinoma.

The pathophysiology of MASLD is closely related to the components of metabolic syndrome: obesity, type 2 diabetes mellitus, dyslipidemia, and insulin resistance [7,9,12,13,14,15,16,17,18]. The classic “two-hit” hypothesis, which assumed a sequential effect of steatosis and oxidative stress, has evolved into a more complex concept of “multiple parallel hits” [19,20,21,22]. This model recognizes the simultaneous and synergistic contribution of multiple pathophysiological factors to disease progression. Key components of this process are lipotoxicity caused by excessive accumulation of free fatty acids, oxidative stress, and associated mitochondrial dysfunction [23]; endoplasmic reticulum (ER) stress, proinflammatory cytokines secreted by both liver cells and visceral adipose tissue, and gut microbiota dysbiosis [19,21].

Despite the scale of the problem, the possibilities for targeted pharmacotherapy of the disease are extremely limited, with many potential drugs showing promising preclinical efficacy for MASLD therapy in animal models but failing to produce meaningful clinical results in humans [24,25]. One of the main reasons for this is the lack of preclinical models that have been properly validated and fully reflect the complex biology and heterogeneity of the disease in humans, including key metabolic abnormalities and histological features [24]. Animal models have provided valuable insights into the disease, but interspecies differences in liver metabolism, genetics, and disease progression limit the transferability of results [26,27]. In addition, MASLD in humans is typically a slowly progressive disease that develops over many years and has numerous links to other comorbidities that cannot be fully replicated in animal models. In vitro models using human liver cells have become indispensable tools due to their controllable conditions, reduced cost, ethical acceptability, and greater “human relevance” [26,27,28,29,30]. Cell models allow for the simulation of individual links in the disease chain, which can be useful for studying specific mechanisms. Over the past decade, the landscape of in vitro MASLD modeling has evolved, moving from traditional two-dimensional (2D) monocultures of immortalized hepatocyte lines to advanced three-dimensional (3D) platforms, including co-cultured spheroids, liver organoids, and microphysiological “organ-on-a-chip” systems [27,29,30].

The aim of this narrative review is to provide a comprehensive and critical analysis of MASLD models based on the HepG2 cell line. The paper analyzes experimental protocols for the induction of steatosis, examines the metabolic characteristics and limitations of HepG2 cells, and assesses the physiological relevance of various methods.

In this review, the modern MASLD/MASH nomenclature is used as the preferred terminology. MASLD is defined as steatotic liver disease in the presence of at least one cardiometabolic risk factor, whereas MASH refers to the inflammatory form of MASLD with steatohepatitis [31,32,33]. With the introduction of the term MASLD, the historical terms NAFLD and NASH are now used only when discussing the evolution of concepts and classifications; when presenting the results of original studies in which the authors used these specific terms; and in cases where automatic replacement of the term could distort the original inclusion criteria or the phenotype of the model. The transition to MASLD/NASH terminology does not negate the value of the accumulated literature on NAFLD/NASH. According to recent reviews, the vast majority of patients with confirmed NAFLD meet the criteria for MASLD [34,35,36], so historical data remain relevant overall. However, for preclinical models, it is crucial not only to update the name but also to explicitly indicate which specific part of the pathogenesis the system models. For HepG2, this primarily involves hepatocyte-centric aspects of the disease, rather than all clinical aspects of MASLD as a multifactorial and systemic condition. Therefore, in the analytical sections of this review, we use the terms MASLD/MASH to reflect the current understanding of the model’s pathogenic relevance, while retaining the terms NAFLD/NASH only when describing the design of specific published studies.

## 2. Advantages of MASLD Models on Cell Lines Compared to Animals

### 2.1. Species-Specific Differences in Metabolism

Rodents are the most common laboratory models for many diseases. However, significant differences between rodents and humans in lipid and xenobiotic metabolism reduce the transferability of data from MASLD models. In mice, high-density lipoproteins (HDLs) are the main carriers of cholesterol, whereas in humans, low-density lipoproteins (LDLs) predominate [37,38,39,40]. This difference is due to the absence of the cholesteryl ester transfer protein (CETP) in mice and other rodents, a protein that, in humans, facilitates the transfer of cholesterol esters from HDLs to apolipoprotein B (apoB)-containing lipoproteins (VLDLs (very-low-density lipoproteins) and LDLs) [37,39,41]. This fundamental difference has profound implications for MASLD modeling, since the atherogenic lipoprotein profile characteristic of metabolic syndrome in humans, namely an increase in LDLs and a reduction in HDLs, cannot be physiologically reproduced in standard rodent models. Studies in mice have shown that even when human hepatocytes are engrafted into an immunodeficient mouse, CETP activity is absent unless human Kupffer cells are also transplanted [41]. It is likely that macrophages are the main source of CETP in humans. Single-cell sequencing data available in the Human Protein Atlas confirm that *CETP* expression is significantly elevated in macrophages, particularly in Kupffer cells (resident liver macrophages) [42].

These and other data demonstrate that even complex chimeric models are not always able to fully reproduce human lipoprotein biology. In contrast, in vitro models express human apolipoproteins, lipoprotein receptors, and lipid transport proteins [43,44,45,46], providing a metabolically relevant context for studying lipid accumulation pathways relevant to MASLD.

The expression levels and tissue specificity of patatin-like phospholipase domain-containing protein 3 (*PNPLA3*) also differ between humans and experimental animals. The *PNPLA3* gene encodes a lipase with triglyceride hydrolase and retinyl ester hydrolase activity. The I148M variant (rs738409 C>G) is the strongest common genetic risk factor for MASLD [47,48,49]. There is an important interspecies difference in the expression of the *PNPLA3* gene. In mice, *Pnpla3* is predominantly expressed in adipose tissue, where its expression level is 50–100 times higher than in the liver [50,51,52]. In contrast, in humans, the highest expression of *PNPLA3* is observed in the liver, with lower levels in the skin and adipose tissue [51,52]. At the same time, in the human liver, *PNPLA3* expression in stellate cells (HSCs) is more than twice that in hepatocytes, which is important for understanding the role of this gene in fibrogenesis [52]. This fundamental difference must be taken into account when interpreting results obtained in mouse models and extrapolating them to the pathogenesis of MASLD in humans.

Cytochrome P450 (CYP450) enzyme profiles also differ significantly between species, affecting drug metabolism and hepatotoxicity. The major human isoenzymes of drug metabolism (CYP3A4, CYP2D6, CYP2C9, CYP2C19, and CYP1A2) lack direct orthologs in mice. For example, CYP3A4, which is responsible for the metabolism of about 50% of drugs, is functionally replaced in mice by *Cyp3a11* and *Cyp3a25*, which have different substrate specificity and catalytic efficiency. Similarly, there are at least four homologues for human CYP2C8, CYP2C9, and CYP2C19 in mice, namely *Cyp2c29, Cyp2c38, Cyp2c39*, and Cyp2c55, while CYP2D6 corresponds to the pair *Cyp2d9* and *Cyp2d10* [53,54]. This fundamental interspecies difference must be taken into account when interpreting preclinical studies of xenobiotic metabolism and toxicity conducted in mouse models.

### 2.2. Control Conditions

A key advantage of in vitro models is the ability to precisely and independently control individual variables in a way that is virtually impossible in studies on whole organisms [29,55]. In cell models, it is possible to strictly set and independently vary the concentration and composition of fatty acid mixtures, glucose and insulin concentrations to simulate varying degrees of insulin resistance, oxygen tension, cytokine and chemokine exposure, co-culture configurations (addition of stellate cells, Kupffer cells, or endothelial cells), as well as the effects of potential therapeutic agents in controlled doses [29,30,55].

### 2.3. High-Throughput Screening Capabilities and Scalability

The pharmaceutical industry’s high demand for MASLD models compatible with high-throughput screening (HTS) is one of the strongest arguments in favor of cell line-based models [27,29]. Induction of MASLD in vitro using free fatty acids leads to a pronounced and quantitatively measurable accumulation of lipids in cell lines within 24–72 h [29]. This rate contrasts sharply with animal models; for example, the most commonly used high-fat diet (HFD) induces simple steatosis in mice within 8–16 weeks [56,57]. The development of steatohepatitis with signs of inflammation and ballooning degeneration of hepatocytes usually requires a longer period of 16–36 weeks. To achieve this, Western diets high in saturated fats, cholesterol, and fructose, or the diet used in the DIAMOND model, are used, which allow for these histological changes to be achieved within the specified time interval [24,58]. Fibrosis and, especially, cirrhosis are the final stages, the formation of which requires the longest dietary exposure—usually 24–52 weeks or more [24,29,56,58,59,60]. The compatibility of cell models with standard microplate formats (e.g., 96-well or 384-well) allows for the use of automated dispensing, microscopy, and other technologies, making it possible to screen thousands of compounds in a short period of time [61,62]. Immortalized cell lines can be maintained indefinitely with stable characteristics, which expands experimental possibilities and at the same time provides greater throughput compared to animal models. However, it is important to note that the immortalization process and long-term passage can potentially lead to changes in cell characteristics, requiring careful validation of each specific line [29,63,64,65].

### 2.4. Ethical and Economic Aspects

The ethical requirement to minimize the use of animals in biomedical research is formalized in the internationally recognized 3R principles—Replacement, Reduction, and Refinement—first formulated by Russell and Burch in 1959 and later enshrined in the regulatory frameworks of many countries. In recent years, it has gained additional momentum thanks to initiatives such as the FDA Modernization Act (2022 FDA Modernization Act), which encourages the adoption of alternative methods [66,67,68,69,70,71]. When studying MASLD, where in vitro models have reached a level of complexity sufficient to solve many problems that previously required animal experiments, the principle of Replacement takes on particular significance. The economic advantages of in vitro models over animal modeling, although secondary to scientific and ethical arguments, are also significant.

### 2.5. Systemic Limitations of Animal Models in MASLD Research

The assessment of the advantages of in vitro models should be considered in light of the well-documented limitations of animal models of MASLD [56,58,72]. No animal model reproduces all the key features of human MASLD/MASH simultaneously.

Methionine–Choline-Deficient diet (MCD diet) rapidly induces steatohepatitis and fibrosis within 3–8 weeks, while also causing significant weight loss, reducing plasma triglyceride (TG) and insulin levels, and failing to reproduce the metabolic syndrome phenotype [56]. High-fat diet (HFD) models are closer to metabolic syndrome but usually develop only moderate steatosis and minimal inflammation within 12–16 weeks and rarely progress to significant fibrosis within a reasonable timeframe [58]. Genetically modified models offer partial solutions. Mice with leptin deficiency (ob/ob) and leptin receptor defect (db/db) develop severe obesity, insulin resistance, and hepatic steatosis but rarely progress to steatohepatitis without additional dietary manipulations (e.g., combining ob/ob with an MCD diet). In addition, leptin deficiency or resistance is rarely found in patients with obesity or MASH, which limits the application and interpretation of data obtained from these models [58]. These characteristics partly explain the failure of clinical trials despite success in preclinical trials on animal models. In real clinical settings, patients with MASLD often have comorbidities and several independent risk factors, reflecting the common pathophysiological basis of these conditions within the metabolic syndrome [73,74]. The most significant comorbidities in MASLD include type 2 diabetes mellitus, cardiovascular disease, chronic kidney disease, and obstructive sleep apnea [73,75,76,77]. In this regard, patients often take several drugs simultaneously to correct these conditions, many of which can themselves affect liver metabolism. Such pharmacological and metabolic complexity is virtually impossible to reproduce in any single animal model, whereas in vitro models allow for the systematic modeling of specified combinations of metabolic stressors and drug exposures in a controlled design.

## 3. Human Liver Cell Lines for Modeling NAFLD

### 3.1. Characteristics and Application of the HepG2 Cell Line for Modeling MASLD

HepG2 is an immortalized human liver cell line isolated from the liver tumor tissue of a 15-year-old male patient. HepG2 is one of the most popular human cell lines for modeling NAFLD [29]. Although HepG2 is commonly classified as hepatocellular carcinoma, modern molecular cytogenetic data classify it as a derivative of hepatoblastoma (HB), which is confirmed by characteristic abnormalities in the Wnt/β-catenin signaling pathway and genetic features typical of HB [78].

An important feature is the presence of a homozygous genotype for the *PNPLA3* I148M (rs738409 C>G) variant, the strongest genetic predictor of MASLD susceptibility and progression. This feature gives the model a number of specific phenotypic traits that are important for preclinical studies, since the presence of the mutant allele causes an increased tendency to steatosis in response to free fatty acid loading [79,80]. As demonstrated by Chen et al., HepG2 (148M/M) cells are significantly more sensitive to PA-induced lipotoxicity and ER stress-mediated apoptosis compared to wild-type cells. This effect is achieved through hyperactivation of the PERK/eIF2α/CHOP signaling pathway and is independent of the production of the lipoapoptotic mediator LPC (lysophosphatidylcholine) [80]. These characteristics make the HepG2 line a sought-after model for studying PNPLA3-dependent molecular mechanisms of steatosis and lipotoxicity.

HepG2 is characterized by pronounced aneuploidy and changes in the number of genome copies (copy number variations, CNVs). According to whole genome sequencing (WGS) data, the line is in a hyperdiploid state with a mosaic genome structure: about 74% of the genome has a baseline copy number (CN) of two, while significant proportions are accounted for by regions with CN = 3 (15.5%) and CN = 4 (2.7%), and there are also segments with CN = 1 and CN > 4. A critically important feature is the presence of extensive regions of loss of heterozygosity (LOH), particularly on chromosomes 6, 11, 14, and 22 [81]. These genomic features must be taken into account when using HepG2 as a model in toxicological and metabolic studies and for the correct interpretation of CRISPR experiments and allele-specific expression [81,82].

### 3.2. Metabolic Profile, Advantages, and Limitations of the HepG2 Cell Line for Modeling MASLD

HepG2 has unlimited growth, is easy to cultivate and cryopreserve, and retains a number of key hepatic phenotypic characteristics, such as epithelial morphology, albumin expression, urea and glycogen synthesis, and the uptake and storage of exogenous fatty acids and lipoprotein remnants [29]. This model is widely used to study lipid accumulation. When treated with a mixture of free fatty acids (e.g., oleic acid (OA) + palmitic acid (PA)), HepG2 cells show significant triglyceride accumulation [83,84,85]. HepG2 gives a predictable steatogenic response to free fatty acids (FFAs) and/or carbohydrate overload. This makes the cell line convenient for standardized comparative analysis of various experimental parameters, such as dose, time, and nutrient combinations, for medium- and high-throughput screening and parallel endpoint panels (lipid droplets, reactive oxygen species (ROS), ER stress, apoptosis, transcriptional responses) [83,86,87,88,89]. The advantages of the MASLD model in this cell line include reproducibility, preservation of key lipid metabolism pathways, high transfection efficiency, suitability for high-throughput screening (HTS), rapid lipid accumulation upon exposure to SFA, low cost, etc. [27,29,79]. HepG2 is compatible with a wide range of methods and areas of study, such as small interfering RNA (siRNA)/CRISPR approaches, the study of the relationship between stress responses and inflammatory pathways, including NLRP3-associated events under PA load [90], and the study of metabolic changes in biochemistry and molecular biology [78], which allows for the study of phenotypes and disease mechanisms (regulation of β-oxidation, inflammatory cascades) in the same cell system [80,91,92,93,94]. Thus, HepG2 is suitable for primary testing of intracellular TG and lipid droplet reduction, normalization of ER stress/ROS markers, restoration of mitochondrial function in lipotoxicity, and effects on transcriptional programs (lipogenesis, β-oxidation, inflammation) [80,91,92,93,94]. In addition, this cell line is convenient for simultaneously monitoring viability and cytotoxicity markers to avoid mistaking the “anti-steatotic” effect for a consequence of cell death [95,96,97].

Despite these obvious advantages, there are also limitations, such as tumor origin. This means that responses to nutrients, certain hormones, and other factors in HepG2 may be skewed relative to normal hepatocytes, which may affect the data on the potency of the substances being studied. In addition, HepG2 is characterized by a metabolic shift known as the Warburg effect [98,99]. This phenomenon consists of the preferential use of glycolysis for energy production even in the presence of oxygen, accompanied by increased glucose consumption, increased lactate production, and relative suppression of oxidative phosphorylation (OXPHOS) [98]. In HepG2 cells, this is confirmed by the increased expression of key glycolysis genes such as *ENO1*, *PKM2*, and *ERR-gamma*, as well as a decrease in OXPHOS activity, as evidenced by a decrease in adenosine triphosphate (ATP) production [98]. It is important to note that mitochondrial function in HepG2 is not completely lost, as OXPHOS can contribute to energy balance, and its activation; for example, under the action of metformin, it can suppress glycolysis and enhance apoptosis [98,99]. Along with this, the HepG2 cell line shows functional weakening of mitochondrial β-oxidation of fatty acids. Studies show that HepG2 mitochondria are vulnerable to the effects of saturated fatty acids, which leads to a decrease in the activity of respiratory chain complexes and ATP production [100]. Moreover, basal β-oxidation activity in HepG2 is under strict negative control, such as due to the protease CLPX (Caseinolytic Mitochondrial Matrix Peptidase Chaperone Subunit X), and it can be significantly induced (2–3 times) by knocking out the corresponding gene or by stimulation with glucagon [101].

HepG2 is characterized by a sharply reduced expression of Phase I biotransformation enzymes— CYP450 —compared to primary human hepatocytes [102,103,104,105]. For example, mRNA levels of most key isoforms, including CYP1A2, CYP2C9, CYP2C19, CYP2D6, CYP2E1, and CYP3A4, in HepG2 cells can be hundreds of times lower than in freshly isolated or short-term cultured hepatocytes [102]. This is not due to post-transcriptional events but to reduced transcriptional activity of CYP450 genes, which in turn correlates with an altered expression profile of hepatic transcription factors such as HNF-4α, C/EBP, and HNF-3 [102]. Although some isoforms (e.g., CYP1A1) retain their ability to induce, the basal activity of xenobiotic metabolism in HepG2 remains extremely low [103]. This limitation must be taken into account when using this cell line as a model for studying drug metabolism and toxicity, as it may lead to false negative results for compounds requiring metabolic activation.

A key feature of Phase II biotransformation in HepG2 cells is that the expression of most conjugating enzymes is better preserved than that of CYP450. In particular, the levels of the sulfatases SULT1A1, SULT1A2, SULT1E1, microsomal glutathione-S-transferase (mGST-1), N-acetyltransferase 1 (NAT1), and epoxide hydrolase (EPHX1) in HepG2 differ only slightly from those in cryopreserved primary human hepatocytes (PHHs), whereas CYP450 activity is significantly reduced [106,107].

The profile of conjugating enzymes in HepG2 is uneven. The expression of *SULT1A3* and *SULT1A4* in HepG2 cells shows relative overexpression—up to 4 times higher than in PHHs [108]. At the same time, levels of UGT1A1 and UGT1A6 may be 10–1000 times lower than in PHHs [106]. The UGT system as a whole is characterized by fragmentary preservation, since HepG2 does not reproduce the full hepatic profile of the UGT1A cluster, which is significantly better represented in HepaRG cells [108]. At the same time, individual isoforms, such as *UGT2B10* and *UGT2A3*, and in some transcriptomic datasets also *UGT2B11* and *UGT2B28*, are expressed in HepG2 at levels close to those in PHHs [108]. From a practical standpoint, this means that glucuronidation in HepG2 is selective in nature.

Certain GST isoforms, including GSTP1, may be maintained in HepG2 cells at levels comparable to those in the liver and PHHs [108]. For example, according to transcriptomic analysis, *GSTP1* expression in HepG2 is comparable to that in the liver [108]. This makes the cell line suitable for studying the cellular antioxidant response and glutathione-dependent detoxification but does not guarantee a physiologically accurate reproduction of the entire adult human liver GST pool. Thus, in HepG2, Phase II biotransformation is partially preserved and, on average, better than Phase I, but it is qualitatively shifted.

Recent reviews and experimental studies directly interpret HepG2 as a model suitable for solving certain toxicological problems, in particular for studying general mechanisms of cytotoxicity and cellular stress response (e.g., activation of transcription factors activator protein-1 (AP1), p53, nuclear factor-erythroid 2 related factor 2 (Nrf2), and nuclear factor kappa B (NF-κB)) [109,110,111], but limited for quantitative prediction of the metabolism and biotransformation of many compounds [109,110,111,112,113]. To overcome this limitation, either metabolic activation systems (e.g., addition of microsomes or S9 (post-mitochondrial supernatant) fraction) [109,111] or genetically modified HepG2 lines with stable overexpression of individual CYP450 isoforms are utilized [112,113]. In addition, HepG2 is characterized by low secretion of VLDLs [114]. Unlike normal hepatocytes, which secrete ApoB100 as part of triglyceride-rich VLDL particles [115], HepG2 cells show incomplete lipidation of ApoB100. As a result, they secrete predominantly denser “lipid-poor” particles corresponding in density to low-density lipoproteins (LDLs), and only a very small fraction (<5%) in the form of mature VLDL particles [114].

One of the key metabolic differences between hepatocellular carcinoma cells, including the HepG2 line, and normal human primary hepatocytes is the expression of different isoforms of hexokinase, an enzyme that catalyzes the first step of glycolysis [116,117,118]. While mature hepatocytes express glucokinase (GCK, hexokinase IV), whose activity is regulated by physiological glucose concentrations and insulin, GCK expression is suppressed in HepG2 cells, which produce high-affinity hexokinase II (HK2) [116,117,118]. HK2 has a higher affinity for glucose and is capable of maintaining a high rate of glycolysis even at low substrate concentrations, which is an integral part of metabolic reprogramming in tumor cells [117]. Moreover, it has been shown that replacing HK2 with GCK in hepatoma cells is not capable of restoring their proliferative potential and tumorigenicity, which emphasizes the specific role of HK2 in carcinogenesis [117].

HepG2 cells are characterized by reduced sensitivity to insulin (insulin resistance), which has been demonstrated in numerous studies using models induced by tumor necrosis factor-α (TNFα), high glucose concentrations, or prolonged insulin stimulation [119,120,121]. Under these conditions, there is a reduction in the phosphorylation of key components of the insulin signaling pathway, including the insulin receptor (IR), IRS-1 substrate, and Akt kinase, leading to impaired insulin-stimulated glucose uptake and glycogen synthesis [119,120,121].

The limited reproducibility of fructose-induced DNL is a particularly significant limitation for MASLD studies, where sugar/fructose loading is considered one of the key pathogenic factors, as the ability of fructose to induce steatosis directly in HepG2 hepatoma cells remains controversial. In a number of studies, despite the use of physiological and even excessive concentrations (up to 20–25 mM) and various incubation times (from 48 h to 28 days), there has been no significant increase in triglyceride content or expression of key de novo lipogenesis genes (*FASN*, *ACACA*) under the influence of fructose alone [91,122,123]. In some cases, high-fructose concentrations (50–80 mM) even led to a decrease in triglyceride levels [122]. These data contrast with studies where a steatogenic effect is observed with the combined exposure of fructose and saturated fatty acids, such as palmitate [124]. The absence of the expected steatogenic response in HepG2 may be due to the metabolic characteristics of this cell line, in particular the low expression of aldolase B and the predominance of the glycolytic phenotype [122], as well as the fact that the basal glucose level in the medium may negate the effect of fructose [123]. Thus, when interpreting the results of studies on the HepG2 model, it is necessary to take into account that fructose does not always act as an independent inducer of lipid accumulation [91,122,123]. Furthermore, the result depends on specific culture conditions and may be reproducible when research protocols are optimized, and differences can be explained by clonal drift between laboratories. In this regard, a promising area of research is the comparison of fructolytic enzyme expression in HepG2 cells of different passages. In addition to metabolic characteristics, the use of the HepG2 model is associated with a number of conceptual limitations. First, like any hepatocyte monoculture, this system does not contain non-parenchymal liver cells—Kupffer cells, endothelial cells, and stellate cells. Their absence precludes the possibility of modeling intercellular communication, which plays a key role in the development of inflammation and fibrogenesis in vivo. Second, to induce steatosis, supraphysiological concentrations of FFAs (concentrations of 0.5–2.0 mM) are often used in HepG2 experiments [28,91,125,126,127,128,129,130]. These limitations are systematically discussed and serve as a basis for the transition to 3D or microphysiological systems [29].

Nevertheless, HepG2 is a practical and methodologically convenient model for reproducing early stages of MASLD, such as steatosis, lipotoxicity, mitochondrial dysfunction, and ER stress, as well as for primary pharmacological screening, taking into account the existing limitations. The most appropriate strategy for using HepG2 in MASLD research is to treat it as a model of hepatocyte-centric steatosis/stress responses and, for questions of progression to MASH/fibrosis and for rigorous DMPK/DILI tasks (Drug Metabolism and Pharmacokinetics (DMPK)/Drug-Induced Liver Injury (DILI)), to supplement it with more physiological systems (HepaRG/PHHs, 3D/chips, co-cultures) and/or engineered derivative lines. Table 1 summarizes the key metabolic characteristics of HepG2 in comparison with PHH and HepaRG cells, highlighting the fundamental differences that must be considered when interpreting experimental data.

### 3.3. Analysis of MASLD Models in HepG2 Cells

Based on the analysis of modeling techniques presented in the literature, a hierarchical classification of models can be distinguished according to increasing complexity, each of which reproduces different aspects of MASLD pathogenesis [28,93,100,126,127,128,131,132,133,134,135,136]. It should be emphasized that, for HepG2-based in vitro systems, it is more accurate to speak not of a complete reproduction of the MASLD nosological entity but rather of the modeling of MASLD-associated hepatocyte-mediated pathways in pathogenesis—steatosis, lipotoxicity, ER stress, mitochondrial dysfunction, and proinflammatory signals. This is because the clinical definition of MASLD, as previously noted, includes not only the presence of steatosis but also a cardiometabolic context [31,32,33], which cannot be fully reproduced in a monoculture of hepatocyte-like cells.

#### 3.3.1. 2D Models Based on HepG2

2D monoculture is the most common and methodologically simple approach. HepG2 cells are cultured in a standard monolayer on plastic and exposed to free fatty acids and/or carbohydrates. This simulates steatosis, lipotoxicity, mitochondrial dysfunction, and ER stress. A classic example is one of the earliest studies—a model of steatosis using OA, where lipid accumulation in HepG2 develops in a dose-dependent manner and can be quantified with high reproducibility using a colorimetric modification of the Oil Red O approach [93]. In subsequent studies, in vitro modeling of liver steatosis in the HepG2 cell line was performed by incubating cells with a mixture of free fatty acids, most often OA and PA, in a molar ratio of 1:1 or 2:1, at a total concentration of 0.5 to 1 mM, for 24–48 h [28,125]. There are different variations in the experiment design; for example, in the work of Arruda et al., NAFLD was modeled in vitro on the HepG2 cell line by incubating cells for 24 or 48 h with free fatty acids—OA and PA—conjugated with 4.5% bovine serum albumin (BSA), in three variants: palmitic acid alone (0.7 mM) or a mixture of both acids in a 2:1 ratio (OA:PA) at a total concentration of 1 mM and 2 mM. The main objectives of the methodology were to comprehensively characterize the model, including the assessment of dose-dependent lipid accumulation, induction of lipotoxicity, oxidative stress, and proinflammatory response [125]. In the study by Gómez-Lechón et al., when modeling steatosis, the HepG2 cell line was incubated with a mixture of free fatty acids—OA and PA in various ratios (3:0, 2:1, 1:1, 1:2, 0:3) and concentrations (0.5, 1, 2 mM) for 24 h. The authors found that a 2:1 OA/PA ratio at a total concentration of 2 mM induces significant lipid accumulation with minimal toxicity, modeling steatosis, while a 0:3 ratio (palmitate only) causes a pronounced cytotoxic and apoptotic response, mimicking acute lipotoxic damage [126].

Nie et al. created a model of steatosis and inflammation in the HepG2 cell line corresponding to NASH (advanced stage of NAFLD) by treating cells for 24 h with a mixture of free fatty acids—OA and PA—at concentrations of 0.5 mM and 0.25 mM, respectively [136]. The aim of the experiment was to induce intracellular lipid accumulation and oxidative stress to create a relevant platform for evaluating the therapeutic effects of exosomes, in particular their ability to modulate fatty acid metabolism and reduce the expression of proinflammatory markers.

In the work of Cui et al., in vitro modeling of hepatic steatosis was performed on the HepG2 cell line using OA at concentrations ranging from 0.1 to 2.0 mM for 24 h to induce lipid accumulation. The key method for quantitative assessment of steatosis was a colorimetric assay developed by the authors based on Oil Red O staining, which allows for an accurate measurement of the degree of lipid accumulation by optical density at a wavelength of 405 nm. In the absence of exogenous inflammatory mediators, OA-induced steatosis was associated with increased production of tumor necrosis factor-α (TNF-α), decreased *PPARα* (peroxisome proliferator-activated receptor alpha) expression, increased lipid peroxidation, apoptosis (through caspase-9 activation), and suppression of cell proliferation (through increased p27). The aim of the study was to develop a convenient, reproducible quantitative model of steatosis for studying direct pathogenic changes in hepatocytes in NAFLD and screening potential therapeutic interventions [93].

In the study performed by Torabi et al., NAFLD was modeled in vitro using the HepG2 cell line by treating cells for 24–48 h with saturated palmitic acid (0.7–0.8 mM), monounsaturated OA, or a combination of both in DMEM/F12 medium supplemented with GlutaMAX containing bovine serum albumin (BSA). The effective concentration and ratio of acids were determined after 24 and 48 h using MTT analysis [127]. The experiment was aimed at quantitatively assessing the accumulation of lipid droplets using Oil Red O staining and analyzing the expression of key pro- (*P53*, *BAX*, *FASL*) and anti-apoptotic (*BCL-2*) genes by real-time polymerase chain reaction (PCR).

In the study by García-Ruiz et al., NASH was modeled in vitro using the HepG2 cell line. To induce mitochondrial dysfunction characteristic of steatohepatitis, cells were treated with saturated fatty acids—PA (200 μM) or stearic acid (200 μM)—for 24 h, while monounsaturated OA (200 μM) was used as a control [100]. The key objectives of the experiment were to evaluate the activity of OXPHOS complexes, ATP content, nitro-oxidative stress levels (3-nitrotyrosination of proteins, NADPH oxidase activity), and mitochondrial DNA stability. The methodology was validated using inhibitors (MnTBAP, VAS2870) and the NADPH oxidase silencing procedure, which confirmed the key role of this enzyme in mediating fatty acid-induced mitochondrial damage.

Bao et al. described a method for creating a MASLD cell model on the HepG2 cell line by treating cells with OA at concentrations ranging from 0.125 to 1.0 mM in dimethyl sulfoxide (DMSO) medium for 24 h [128]. The main 2D steatosis induction protocols discussed above are summarized in Table 2.

Analysis of induction conditions in various studies using the HepG2 cell line reveals the following patterns [28,93,100,126,127,128,131,132,133,134,135,136]. OA (C18:1, monounsaturated) is the main steatogenic agent and, when used in isolation, it induces dose-dependent lipid accumulation with relatively low cytotoxicity. PA (C16:0, saturated) predominantly induces a lipotoxic, proapoptotic, and proinflammatory response. A combination of OA:PA in a 2:1 ratio is recognized as optimal for modeling steatosis with minimal toxicity [126], whereas the use of isolated PA simulates acute lipotoxic damage.

The range of 0.1 to 2.0 mM for the combination of FAs is the standard range for modeling MASLD in 2D HepG2 monoculture [93,100,125,126,127,128]. Higher concentrations (1–2 mM) are used for severe steatosis and lipotoxicity; moderate concentrations (0.2–0.75 mM) are used to study specific molecular mechanisms, such as mitochondrial dysfunction. For example, in the work of García-Ruiz et al. [100], it was shown that adding saturated palmitic acid (200 μM, 24 h) to the HepG2 cell line is sufficient to develop oxidative and nitrosative stress without the need to model the complete phenotype of the disease. The key mechanism of this effect is the activation of NADPH oxidase (NOX), which subsequently causes accelerated degradation of OXPHOS subunits and damage to mitochondrial DNA [100].

Various studies predominantly use 24 h incubation, while 48 h incubation is used to enhance effects or assess temporal dynamics. These conditions ensure intensive lipid accumulation and activation of apoptotic pathways. In 2D models, longer exposures (≥7 days) are rarely used due to the limitations of monolayer culture. Fatty acids are conjugated with bovine serum albumin (BSA, 1–4.5%, or fat-free BSA) to ensure solubility and mimic physiological transport. Alternatively, DMSO is used [128].

#### 3.3.2. HepG2-Based 3D Models

The creation and use of HepG2-based 3D MASLD models aim to overcome the limitations of 2D models, such as the absence of intercellular contacts in three dimensions, non-physiological geometry, and rapid loss of functional phenotype. This review discusses three main 3D platforms based on HepG2 cells [131]. These platforms are summarized in Table 3.

The model developed by Wiriyakulsit et al. [131] on an organ-on-a-chip (OOC) platform demonstrated the possibility of screening drug substances. The study showed that pioglitazone and elafibranor exhibited fundamentally different effect–safety profiles. Pioglitazone improved viability, while elafibranor reduced steatosis but was accompanied by a decrease in functional parameters. This highlights the value of 3D platforms for screening and evaluating the “effect–safety” ratio at the preclinical stage. In this study, NAFLD was modeled in vitro using the HepG2 cell line, cultured in a three-dimensional format on an organ-on-a-chip (the Mimetas OrganoPlate platform) in a type I collagen matrix. Steatosis was induced within 24 h by treatment with free fatty acids—OA (0.5 mM), PA (0.5 mM), or a mixture of both (OA + PA, 0.25 mM each, total concentration 0.5 mM), conjugated with 1% fat-free bovine serum albumin (BSA), in two media variants (Condition A: 10% fetal bovine serum (FBS); Condition B: 0.5% FBS) [131].

Yang et al.’s model (“gut–liver-on-a-chip”) is the most physiologically complex of the platforms described, reproducing the axis of interorgan interaction with closed-loop circulation. Prolonged exposure (7 days) brings the model closer to the chronic pathogenesis of MASLD. MASLD modeling in this study was performed on an integrated microfluidic “gut–liver-on-a-chip” platform with co-cultivation of Caco-2 and HepG2 cell lines. Steatosis was induced by treatment with a mixture of free fatty acids—PA and OA in a molar ratio of 1:2 and a total concentration of 1 mM in a serum-free medium for 1 or 7 days with closed-loop circulation. The aim of the study was to create a physiologically relevant in vitro model of the gut–liver axis to study the role of intertissue interactions in the initiation and progression of NAFLD, as well as to evaluate the effect of lipid load on the cellular response and function of hepatocytes [135].

Despite their significant advantage over 2D models, the described 3D platforms have a number of limitations. Microfluidic-based models, although more physiologically relevant, are technically complex and expensive, while static spheroids are prone to problems with the formation of oxygen and nutrient gradients.

#### 3.3.3. MASLD Modeling Using Co-Cultures

MASLD modeling using co-cultures aims to reproduce the tissue microenvironment of the liver, including intercellular interactions between hepatocytes and non-parenchymal cells (stellate, immune), which is a key factor in the progression from steatosis to steatohepatitis and fibrosis [132,133,134]. Representative co-culture models are presented in Table 4.

In the study by Pingitore et al., steatosis and fibrosis were modeled in vitro on 3D spheroids consisting of a co-culture of HepG2 cell line and the immortalized hepatic stellate cell line LX-2 in a physiological ratio of 24:1, carrying the *PNPLA3* I148M genetic variant. To induce steatosis and fibrogenesis, compact spheroids were incubated in a medium containing 500 μM of a mixture of free fatty acids (PA and OA) [132]. The model by Rafiei et al. (triculture) [134] represents a comprehensive approach. The use of an “activating mixture” of several components (FFAs + glucose + insulin + LPS + TGF-β + BSA) simultaneously mimics the metabolic, inflammatory, and profibrogenic risk factors of MASLD, allowing for the reproduction of steatosis, oxidative stress, inflammation, and fibrogenesis within a single experimental system. Validation of the model confirms its functional suitability.

#### 3.3.4. Comparative Analysis of Modeling Methods

A comparative analysis of three main cultivation formats is provided in Table 5 (traditional 2D monoculture, volumetric 3D models (spheroids, OOC), and multicomponent co-cultures/tricultures) revealed significant differences in their ability to reproduce individual links in pathogenesis, experimental characteristics, and areas of application.

HepG2 2D monocultures remain the most accessible tool for studying the initial stages of cell damage. They allow for the reliable reproduction of key processes such as lipid accumulation (steatosis), lipotoxicity, ER stress, oxidative stress, and mitochondrial dysfunction. The main advantage of 2D systems is their simplicity and the ability to obtain results quickly.

3D models (including spheroids and microfluidic platforms) retain the ability to reproduce all of the above metabolic disorders but add a fundamentally important level of organization—three-dimensional spatial architecture. This provides more physiological intercellular communication, improved cell differentiation, and the possibility of long-term cultivation, which, in turn, opens up prospects for pharmacokinetic studies and the assessment of the chronic effects of drug compounds.

Co-cultures and tricultures are the most complex and physiologically relevant in vitro models. The inclusion of non-parenchymal liver cells (stellate cells, Kupffer cells, endothelial cells) allows for the reproduction of key disease progression processes that are not accessible to simpler systems. These include the production of proinflammatory cytokines (modeling inflammation), activation of stellate cells, and synthesis of extracellular matrix components, in particular type I collagen (modeling fibrogenesis), as well as the study of complex mechanisms of intercellular communication underlying the transition from simple steatosis to steatohepatitis.

**Figure 1:**
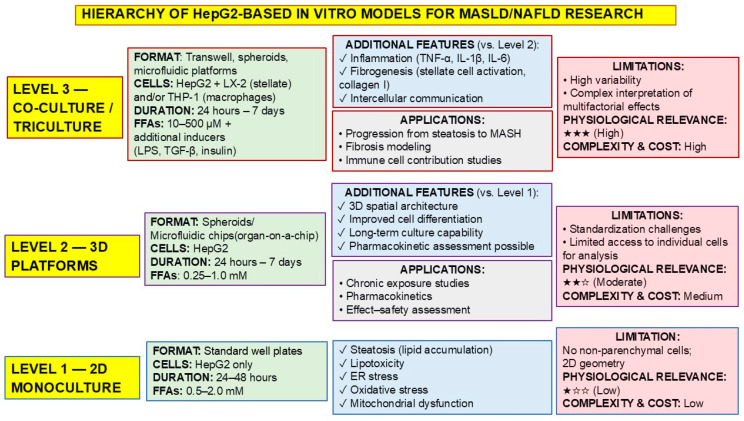
Hierarchy of HepG2 cell models ranked by increasing physiological relevance for studying MASLD. Level 1 (two-dimensional monoculture) reproduces early cellular changes, including steatosis, lipotoxicity, ER stress, oxidative stress, and mitochondrial dysfunction. Suitable for high-throughput screening. Level 2 (3D models)—spheroids and “organ-on-a-chip” platforms—provides spatial organization and improved cell differentiation and enables long-term assessment of culture and pharmacokinetics. Level 3 (co-culture/triculture) includes stellate liver cells (LX-2) and/or macrophages (THP-1), which allows for the modeling of inflammation, fibrogenesis, and intercellular communication, thereby replicating key features of MASLD progression. Physiological relevance, complexity and cost are indicated by stars: ★★★ high, ★★☆ moderate, ★☆☆ low.

The analysis allows us to establish the following hierarchy in ascending order of physiological relevance: low/moderate for 2D monocultures, moderate/high for 3D models, and highest for multicomponent co-cultures (Figure 1). However, increased relevance is accompanied by increased complexity and cost, as well as the emergence of new limitations. The key limitation of 2D models remains the absence of non-parenchyma cells and non-physiological growth geometry. For 3D models, the main challenges are the complexity of protocol standardization and limited access to individual cells for post-experimental analysis. In co-cultures, the high variability in the results and the complexity of interpreting multifactorial effects come to the fore when it is impossible to unambiguously determine the contribution of each cell type to the observed effect. It is important to note that a direct quantitative comparison of 2D, 3D, and co-culture models of MASLD based on HepG2 cells is complicated by methodological heterogeneity across published studies: there are differences in the composition and concentrations of fatty acids, exposure duration, the assay readouts used, and the analytical endpoints. Therefore, rather than a formal analysis of absolute values, a normalized comparison across several domains of physiological relevance is more appropriate: the ability to reproduce steatosis, the preservation of liver function, the reproduction of the inflammatory response, and the reproduction of early fibrogenic changes.

#### 3.3.5. Analysis of the Physiological Relevance of the Fatty Acid Concentrations Used

One of the key methodological issues in modeling MASLD in vitro is the comparability of the FFA concentrations used with physiological levels. The normal concentration of FFAs in human plasma on an empty stomach varies depending on gender, age, and the method of measurement. The reference range for serum is 0.1–0.45 mM for women and 0.1–0.6 mM for men [137,138]. In obesity and metabolic syndrome, the concentration of FFAs in fasting blood plasma reaches 0.6–1.0 mM [139,140].

Under physiological conditions, FFAs circulate in the blood primarily in an albumin-bound state [138,141,142]. However, only the unbound fraction is biologically active and amounts to just 1.6 nM, which is less than 10^−5^ of total FFAs [141]. Accordingly, regarding in vitro modeling, clinical relevance is determined not only by total FFAs but also by the FFA:BSA ratio, as well as by the proportion of FFA that remains unbound and available for membrane transport, enzymatic reactions, and lipotoxic damage. This means that identical nominal FFA concentrations in different studies are not necessarily equivalent in terms of the degree of cellular burden. Seven major binding sites for long-chain FFAs have been identified on the albumin molecule, of which three sites (FA2, FA4, and FA5) exhibit high affinity [143,144]. In healthy human plasma, the FFA:BSA molar ratio is typically in the range of 1:1–3:1, whereas in pathological conditions it may exceed 5:1–6:1 [55,145]. Changes in temperature, conjugation time, and the method of preparation of FFA/BSA complexes alter the availability of fatty acids and, consequently, the biological effect. Therefore, when planning an experiment, physiological relevance should be assessed not by total FFAs in isolation but by four variables simultaneously: fatty acid composition, total FFAs, the FFA:BSA ratio, and duration of exposure.

The choice of FFA composition in disease modeling must also be pathogenetically justified. In the postprandial period, higher levels of circulating FFAs in patients with MASLD are largely attributable to oleic and palmitic acids, as well as linoleic acid [146]. At the same time, lipid analysis in patients with MASH shows that palmitic, stearic, and oleic acids remain the main components of the circulating FFA pool [147]. This makes mixtures of OA and PA pathogenetically more justified than the use of a single randomly selected FFA but at the same time emphasizes that even such a mixture reproduces only a portion of the patient’s actual plasma lipid composition.

From a biological perspective, it is important to distinguish between the steatogenic and lipotoxic dosage ranges. Oleic acid primarily promotes the formation of larger lipid droplets and facilitates the safe esterification of fatty acids into triglycerides [148,149,150,151], whereas palmitic acid is more strongly associated with mitochondrial fragmentation, increased oxidative metabolism, and ROS formation in both the cytosol and mitochondria. ROS accumulation triggers damaging cascades: a decrease in mitochondrial membrane potential, activation of caspases 3/7, apoptosis, and ER stress and inflammation via NF-κB [90,152,153,154,155]. Thus, OA or OA:PA mixtures in a 2:1 ratio under moderate loads better correspond to the “simple steatosis” phenotype, whereas increasing the PA fraction or total FFA concentration shifts the model toward the stress response phenotype characteristic of the transition to MASH. In other words, the same total FFA concentration, depending on the proportion of saturated fatty acids in the mixture and albumin binding parameters (FFA:BSA ratio), can lead either predominantly to neutral triglyceride accumulation or cause pronounced lipotoxicity.

From a practical standpoint, the concentration ranges can be interpreted as follows: to model the early stage of steatosis (lipid accumulation without pronounced cytotoxicity or apoptosis), low and moderate concentrations of free fatty acids (FFAs) are typically used, ranging from near-physiological levels (0.1–0.4 mM) to moderately elevated levels (0.75 mM). Thus, in HepG2 cells, treatment with OA at concentrations of 0.125–0.25 mM induces significant triglyceride accumulation without a decrease in viability, whereas concentrations ≥ 0.5 mM already exhibit signs of toxicity [126,128,156].

Moderate and high concentrations of FFAs in 2D HepG2 monocultures should be viewed as a trade-off that reduces modeling time but results in increased toxicity and reduced direct clinical relevance [83,125,126,157]. Arruda et al. demonstrated that incubation with a mixture OA + PA in a 2:1 ratio (under exposure to high FFA concentrations—1–2 mM for 24–48 h) caused more pronounced lipid accumulation, increased markers of oxidative damage, and a significant rise in IL-1β, IL-6, IL-17, IL-22, and TGF-β. However, it should be noted that 48 h exposure did not result in a proportional increase in steatosis; under this regimen, a significant decrease in cell viability was observed [125]. Consequently, concentrations of approximately 1–2 mM, especially with exposure of 48 h or more, promote lipotoxicity to a greater extent [125].

An important pattern can be observed: the more complex the model system (co-culture, 3D, additional inducers), the lower the required concentrations of FFAs to obtain a relevant phenotype. In the 2D format, supraphysiological concentrations are often used, which is a compromise to compensate for the lack of chronic exposure, tissue microenvironment, and additional pathogenetic factors. Analysis of the data directly indicates that supraphysiological concentrations of FFAs are often used in the methods, which may excessively increase toxicity, and this is systematically discussed as a basis for transitioning to 3D systems (Table 6).

Thus, the stage of MASLD in vivo is not determined solely by FFA concentration. The transition from simple steatosis to MASH in the body depends on the duration of exposure, FFA composition, insulin and glucose concentrations, mitochondrial adaptation, the presence of inflammatory signals, and intercellular communication with macrophages, endothelial cells, and stellate cells. Therefore, in vitro studies can only provide a phenotypic approximation of individual disease mechanisms.

### 3.4. Practical Considerations for HepG2-Based MASLD Models

The analysis revealed that the selection of the optimal experimental model based on the HepG2 cell line depends on the research objective. Given the well-documented metabolic limitations of this line (low CYP450 activity, defective lipoprotein secretion, and a shift in energy metabolism toward aerobic glycolysis), its use must be strictly aligned with the specific research objective.

A review of the literature shows that the optimal range of FFA concentrations depends on the complexity of the model. In 2D monocultures, concentrations of 0.5–2.0 mM are more commonly used, which are higher than physiological values [28,125,126,127,128]. This is a widely accepted compromise that allows for steatosis to be induced in a short amount of time (24–48 h). In more complex systems (3D cultures, co-cultures), lower FFA concentrations (10–500 μM) are required [131,132,133,134], which brings conditions closer to physiological ones and reduces the risk of non-specific cytotoxicity.

In studies aimed at the initial screening of anti-steatotic compounds, it is preferable to use OA (alone) or OA:PA mixtures in a 2:1 ratio at moderate concentrations (0.5–1.0 mM), with mandatory monitoring of cell viability [55,126]. If it is necessary to model the transition to lipotoxicity, not only total FFAs but also the proportion of PA should be increased, while simultaneously assessing ROS, markers of ER stress, and apoptosis [55]. If, however, conditions above the conventionally physiological range or FFA:BSA ratios are used, they must be defined in advance as a stressor rather than a direct equivalent of a specific clinical stage of MASLD. In such cases, subsequent validation of key results in more physiologically relevant systems—3D, co-cultures, HepaRG, or PHHs—is recommended.

It is important to emphasize that HepG2 is most suitable for studying the early, hepatocyte-centric events in the pathogenesis of MASLD: steatosis, lipotoxicity, ER stress, oxidative stress, and mitochondrial dysfunction. For tasks related to disease progression (inflammation, fibrosis), as well as for rigorous studies of drug metabolism and toxicity, HepG2 should be used with caution, preferably as part of more complex systems (co-cultures, 3D platforms) or in combination with alternative models (HepaRG, primary hepatocytes) [158,159,160,161].

Due to the existing limitations of HepG2, methods are being developed to functionally tailor it to specific experimental tasks. In practice, strategies for optimizing HepG2 can be divided into several approaches. The first approach is based on genetic modification of cells to express key biotransformation enzymes, in particular cytochrome P450 isoforms [109]. The second approach involves epigenetic and cultural maturation of cells, which allows for increased expression of nuclear receptors and, consequently, of xenobiotic metabolism enzymes [162]. The third approach involves external metabolic activation using subcellular liver fractions, such as the S9 fraction or microsomes, which allows for the modeling of Phase I and II metabolism [109]. Finally, the fourth approach involves transitioning from monolayer culture to three-dimensional (3D) or hybrid formats (co-cultivation with non-parenchymal liver cells) [109,163]. This approach preserves the key advantages of HepG2—reproducibility, ease of cultivation, and compatibility with HTS—while minimizing the risk of incorrect extrapolation of data to the physiology of normal hepatocytes [109].

The most extensively studied approach involves the creation of genetically engineered HepG2 cell lines that express biotransformation enzymes [113]. Stable HepG2 panels with overexpression of individual CYPs have already demonstrated their suitability for identifying metabolically mediated toxicity and for determining the contribution of a specific isoform to the biotransformation of a xenobiotic [112,113]. More complex knock-in/CRISPR-derived HepG2 lines have also been developed, simultaneously expressing CYP1A2, CYP2C9, CYP2C19, CYP2D6, CYP3A4, POR, and UGT1A1. In such cells, the activity of the corresponding enzymes increased significantly and was maintained in later passages, making them useful for specific tasks in metabolic research [164]. A more physiological, though less standardized, approach involves modulating regulatory networks rather than directly introducing a single CYP. For example, lentiviral expression of HNF1α in HepG2 cells increased CYP3A4 activity, preserved the system’s sensitivity to rifampicin induction, and, under optimal transduction conditions, brought activity close to that of primary hepatocytes; however, at high multiplicity of infection (MOI), it was accompanied by increased cell death [165].

Epigenetic reprogramming using 5-azacytidine, vitamin C, and hormonal supplements (insulin and hydrocortisone) partially shifts HepG2 cells toward a more differentiated hepatic phenotype and increases the expression of certain CYP450 enzymes (CYP1A2, CYP2C9, CYP3A4), but enzymatic activity increases only slightly (statistically insignificant) and, as a rule, does not reach PHH levels [162]. Similarly, inhibition of DNA methyltransferase 1 (DNMT1)/double-stranded RNA-dependent protein kinase (PKR) using zebularine can significantly induce a number of CYP450 enzymes (including CYP1A1, 1A2, 2A6, 2B6, 2C9, 2C19, 2D6, 2E1, 3A4) in HepG2 cells, accompanied by increased sensitivity to drug toxicity mediated by CYP450-dependent biotransformation [166]. A HepG2-DP cell line with stable double knockout of DNMT1 and PKR was established, characterized by increased expression of CYP3A4 and albumin and the formation of functional bile ducts, confirming the key role of these targets in the regulation of hepatospecific functions [167].

Of particular interest is metabolic maturation achieved by modifying the nutrient medium. Under amino acid-enriched conditions, a metabolically active HepG2 (mHepG2) variant has been described that exhibits activity of eight drug-metabolizing CYPs (CYP1A2, 2A6, 2B6, 2C8, 2C9, 2D6, 2E1, and 3A4), as confirmed at the transcriptional, proteomic, and metabolic levels. However, this system requires a lengthy preparation period (22 days of cultivation) and does not eliminate the line’s tumorigenic genetic background [168].

For studies in which the formation of reactive metabolites is critical but long-term reproduction of the MASLD phenotype is not required, combined systems of exogenous metabolic activation—primarily liver microsomes and S9 fractions—are appropriate. Microsomes are convenient for CYP450-dependent biotransformation, are relatively low-toxicity, and are well-suited for HTS; however, they are deficient in Phase II enzymes and lack cytosolic conjugation enzymes (sulfotransferases, glutathione-S-transferases, etc.), which makes them less suitable for studying the full spectrum of detoxification reactions [109]. In contrast, S9 fractions contain both microsomal (including cytochromes P450) and cytosolic (glutathione-S-transferases, sulfotransferases) enzymes, which provides a more complete metabolic profile in vitro. However, as noted in the literature, S9 fractions exhibit pronounced cytotoxicity toward cultured cells, which requires additional steps to wash the cells after metabolic activation. Furthermore, the S9 fraction does not replicate key aspects of living cell physiology, such as membrane transport of substrates and nuclear regulation of metabolic enzyme expression [109]. Therefore, such hybrid approaches are particularly useful for short-term tests of metabolically mediated toxicity but have a number of limitations.

Spatial functionalization of the model yields mixed but significant results. In a number of studies, 3D HepG2 spheroids demonstrated increased secretion of albumin and apolipoproteins, as well as enhanced metabolic functions compared to 2D monocultures [169,170]. In this regard, a stepwise strategy appears most rational: use 2D HepG2 models for rapid screening of steatosis and stress responses; use modified HepG2 models (genetically engineered or microsomal/S9-enhanced variants) for questions of metabolic activation. The additional use of other cell lines, such as HepaRG, PHHs, 3D spheroids, and co-cultures, is recommended to validate results when assessing the impact on metabolic pathways, inflammation, fibrogenesis, or the role of intercellular communication. To improve reproducibility, publications must specify the passage number, functional maturation conditions, the format of external CYP450 metabolic activation, and other cultivation details.

Thus, HepG2 serves as a valuable tool for studying certain aspects of MASLD, particularly in the early stages of pathogenesis. However, its limitations (low CYP450 activity, defective VLDL secretion, metabolic shift toward glycolysis) require a cautious approach to interpreting results and, if necessary, supplementation with studies using alternative models (HepaRG, primary hepatocytes, 3D systems, co-cultures). The choice of a specific model should be determined by the specific research objective and supported by appropriate validation.

## 4. Conclusions

MASLD is a clinically and pathophysiologically complex disease that progresses over many years and involves various liver cell types. Hepatocytes play a key role in the pathogenesis of the disease and are therefore the primary focus of research. The use of HepG2 cell cultures is a widely adopted practice for modeling MASLD and offers numerous advantages over animal models. At the same time, there are a large number of different modeling techniques that have undergone a certain evolution: from simple 2D models to more complex 3D models and co-cultures.

Based on an analysis of the descriptions in the literature of methods for modeling MASLD using the HepG2 cell line, the described approaches can be ranked by validity depending on the research objective. For screening and initial assessment of anti-steatotic effects, the most suitable method remains a 2D monoculture with OA:PA in a 2:1 ratio at a concentration of 0.5–1.0 mM for 24 h [126], which provides an optimal balance between steatogenesis and cell viability, high reproducibility, and ease of use for high-throughput screening (HTS). To study the progression of steatosis to steatohepatitis with inflammation and fibrogenesis, a three-cell culture model—such as HepG2 + LX-2 + THP-1 with an activating mixture [134]—represents the most comprehensive solution, simultaneously reproducing steatosis, oxidative stress, inflammation, and fibrosis within a high-throughput system. For pharmacological “effect–safety” evaluation, 3D organ-on-a-chip platforms [131] provide a unique opportunity to simultaneously assess the therapeutic efficacy and toxicity of candidates, which is unattainable in 2D. For genetic studies of PNPLA3-dependent mechanisms, HepG2 + LX-2 3D spheroids [132] utilizing endogenous PNPLA3 I148M homozygosity serve as a unique platform.

Thus, the selection of the optimal MASLD model using HepG2 cells must take into account the metabolic characteristics of these cells and be tailored to the specific research objectives, and the results obtained must be interpreted with an awareness of the model’s known limitations.

## Figures and Tables

**Table 1:** Key metabolic characteristics of HepG2 compared to PHHs and HepaRG.

Parameter	HepG2	PHHs/HepaRG
CYP450 activity	Reduced; weak response to classic inducers	High; physiological inducibility
Hexokinase/glucokinase	Hexokinase II (instead of glucokinase)	Glucokinase (physiological)
VLDL secretion	Defective; incomplete ApoB100 lipidation; denser, “lipid-poor” particles are secreted	Physiological assembly and secretion of VLDLs
Energy metabolism	Warburg effect (aerobic glycolysis)	Predominantly OXPHOS
Insulin sensitivity	Reduced	Preserved
β-oxidation of fatty acids	Reduced expression of a number of key enzymes	Physiological level
Response to fructose (DNL)	Limited reproducibility; fructose does not always enhance steatogenic responses	A more reproducible response

**Table 2:** Protocols for inducing steatosis in a 2D HepG2 monoculture.

Author	FFAs, Composition	Concentration, mM	Time
Cui et al. [93]	OA (mono)	0.1–2.0	24 h
Bao et al. [128]	OA (mono)	0.125–1.0	24 h
Gómez-Lechón et al. [126]	OA:PA	0.5, 1, 2	24 h
Arruda et al. [125]	PA (mono) or OA:PA 2:1	0.7 (PA); 1–2 (total)	24–48 h
Torabi et al. [127]	PA, OA or combination	0.7–0.8 (PA)	24–48 h
Nie et al. [136]	OA + PA	OA 0.5; PA 0.25	24 h
García-Ruiz et al. [100]	PA or stearin (separately), OA—control	0.2 each of the FFAs	24 h

**Table 3:** HepG2-based MASLD 3D models.

Author	Type of 3D Platform	FFAs, Composition	Concentration, mM	Time
Wiriyakulsit et al. [131]	Organ-on-a-chip, collagen I	OA, PA or OA + PA + 1% BSA	0.5 (mono) or 0.25 + 0.25	24 h
Yang et al. [135]	Microfluidics “gut–liver-on-a-chip” (Caco-2 + HepG2)	PA:OA 1:2, serum-free medium	1	1 or 7 days
Pingitore et al. [132]	3D spheroids (HepG2 + LX-2, 24:1)	PA:OA 1:2 + 1% BSA; + transforming growth factor β (TGF-β) 10 ng/mL, platelet-derived growth factor (PDGF) 10 ng/mL	0.5	24–48 h

**Table 4:** HepG2-based MASLD co-culture models.

Author	Cellular Composition	Inductor	Concentrations	Time
Kim et al. [133]	HepG2 + THP-1 (10:1 or 10:5) in gelatin methacrylate (GelMA) 3.5%, transwell	OA:PA 2:1 + 10% BSA	OA 10, PA 5 μM	7 days
Pingitore et al. [132]	HepG2 + LX-2 (24:1), 3D spheroids	PA:OA 1:2 + BSA; TGF-β; PDGF	500 μM FFAs	24–48 h
Rafiei et al. [134]	HepG2 + LX-2 + THP-1 (10:10:1), transwell (triculture)	OA + PA + glucose + insulin + lipopolysaccharide (LPS) + TGF-β + BSA	OA 100 + PA 25 μM + glucose 11 mM + insulin 10 nM + LPS 10 ng/mL + TGF-β 3 ng/mL	72 h

**Table 5:** Comparative analysis of formats: 2D, 3D, and co-culture.

Criterion	2D Monoculture	3D (Spheroids, Chips)	Co-Culture/Triculture
Reproducible links in pathogenesis	Steatosis, lipotoxicity, ER stress, oxidative stress, mitochondrial dysfunction	+ spatial organization, improved differentiation, long-term cultivation, pharmacokinetic evaluation	+ inflammation (cytokines), fibrogenesis (collagen I), intercellular communication
Typical concentrations of FFAs	0.1–2.0 mM	0.25–1.0 mM	10–500 μM (+ additional inductors)
Exposure time	24–48 h	24 h–7 days	24 h–7 days
Use for high-throughput screening (HTS)	High	Average (depends on platform)	Limited (difficulty of staging)
Effect–safety assessment	Limited	Possible (organ-on-a-chip)	Possible
Cost and complexity	Low	Medium–high	High
Physiological relevance	Low–moderate	Moderate–high	The highest of those described in vitro
Key limitation	Absence of non-parenchyma cells; non-physiological geometry	Complexity of standardization; limited access to individual cells	High variability; complexity of interpreting multifactorial effects

**Table 6:** Assessment of the physiological relevance of the concentrations of FFAs used.

Model Category	FFA Concentration Range	Physiological Compatibility	Comment
2D, high doses [125,126]	1–2 mM (total)	Supraphysiological	This approach is justified for 24 h acute exposures to compensate for the lack of chronic stimulation; however, it may lead to a disproportionate increase in toxicity
2D, moderate doses [100,128,136]	0.125–0.75 mM	Close to physiological/moderately elevated levels	Most suitable for studying specific mechanisms
3D/Co-culture [133]	10–15 μM	Sub-physiological	The low FFA range is partly offset by prolonged exposure (7 days) and the 3D culture context, which better approximates chronic disease-relevant metabolic stress
Triculture [134]	OA 100 + PA 25 μM (125 μM total)	Near the lower bound of the physiological range	The effect is potentiated by additional cues (LPS, TGF-β, insulin, and glucose), thereby enhancing inflammatory and profibrogenic signaling despite moderate FFA levels

## Data Availability

No new data were created or analyzed in this study. Data sharing is not applicable to this article.
